# 

**Published:** 1881-05

**Authors:** 


### ASSOCIATION.

The eleventh annual meeting of the Wisconsin State Dental Society will be held in Milwaukee on the third Tuesday in July, 1881, and continue three days.

It is very desirable that every member of the society and dentists from every part of the state should attend this session and contribute to its interest and success.

Judging from letters and circulars that have come to hand within the last few months; this society has some work pertaining to educational matters, that ought to receive attention.

The dental profession in Wisconsin has in its ranks many good and strong men, and it is to be hoped that what their hands find to do they will do with their might.

### ASSOCIATION.

The twelfth annual meeting of the California State Dental Association will be held in San Francisco, commencing June 14, 1881, at 10 o'clock a. m., and continue four days. A very interesting programme of subjects is presented for essays and discussion, in which are the following:

Electricity and its Uses in Dentistry.

Cause and Prevention of Dental Caries.

The future status of Dentistry in California.

Alimentation of Tooth Substance.

Intra Dental Splints.

Diseases of Dental Pulps.

Progress of Dentistry.

Does radical treatment of Alveolar Abscess lead to permanent success?

To each of these an essayist is assigned.

This Association has done a great work for the profession and for the people as well. The work of the body compares well with that of such societies in any other part of the country, though many of them are much older. It is greatly to be desired that all of the honorable and efficient members of the profession in the State should co-operate in this work. The work of dental education now calls for the special attention of this Society. A College is being established, for the organization and proper upbuilding of which this Society will be largely responsible; and the position and reputation of the profession in California for the future will depend much upon this institution.

### CONTENTS.

### COMMUNICATIONS.

A MONTHLY MAGAZINE DEVOTED TO THE INTERESTS OF

**THE DENTAL PROFESSION.**

*TERMS REDUCED TO $2.50 PER YEAR. SINGLE COPIES 25C.*

EDITED BY PROF. J. TAFT, D.D.S.

AND

*Published by SPENCER, CROCKER & CO., Ohio Dental Depot.*

The volume commences in January and closes in December.

The REGISTER being issued monthly is always fresh, therefore Indispensable to the practitioner who desires to keep posted up with the new inventions and improvements which are daily developed. Neither expense nor effort will be spared to give value and variety to its contents. Articles will be illustrated with well executed engravings whenever they will add to the interest or assist to explanation.

Its extensive circulation and frequency of issue make it one of the BEST DENTAL ADVERTISING MEDIUMS in the United States.

All subscriptions must begin with January or July.

Local or special notices, five lines or less, will be inserted for$3 each insertion; over five lines, 50 cts. per line.

All advertising bills payable in advance, or at furthest, after the issue of the first number containing the advertisement. All transient advertisements to be paid for in advance.

☞CONTRIBUTIONS SOLICITED.

Exclusively Editorial Communications should be addressed to Editor.

The latest number of the REGISTER may be ordered with or without other goods at any time, and all letters should be addressed to

*SPENCER, CROCKER & CO., Ohio Dental Depot, Cincinnati, O.*

